# *ABCG2* rs2231142 variant in hyperuricemia is modified by *SLC2A9* and *SLC22A12* polymorphisms and cardiovascular risk factors in an elderly community-dwelling population

**DOI:** 10.1186/s12881-020-0987-4

**Published:** 2020-03-17

**Authors:** Jia Liu, Wei Yang, Yun Li, Zhanyun Wei, Xiaojuan Dan

**Affiliations:** 1 Departments of Geriatric Medicine, Xuanwu Hospital, The Capital Medical University, 45 Changchun Street, Xicheng District, Beijing, 100053 China; 2 Departments of Neurology, Xuanwu Hospital, The Capital Medical University, Beijing, China

**Keywords:** Uric acid, *ABCG2*, Polymorphisms, Hypertension, Triglyceridemia

## Abstract

**Background:**

The *ABCG2* rs2231142 single nucleotide polymorphism (SNP) is one of the most significant genetic variants associated with hyperuricemia (HUA) in Asian populations. However, the risk of *ABCG2* rs2231142 variants for HUA could interact with other important HUA risk variants and cardiovascular factors. This study investigated the effects of the combined association among *ABCG2* rs2231142 and multiple HUA genetic variants or cardiovascular risk factors on HUA risk and serum uric acid (sUA) levels in an elderly Chinese population.

**Methods:**

A total of 1206 participants over 65 years old were enrolled in this study. Physical and laboratory examinations were performed for all participants. The *ABCG2* rs2231142, *SLC2A9* rs3733591, and *SLC22A12* rs893006 SNPs were assayed using a standardized protocol. Logistic regression analysis and liner regression were adjusted respectively to account for the association between *ABCG2* rs2231142 and other genetic variants, as well as between cardiovascular risk factors and HUA risk and sUA levels.

**Results:**

The prevalence of HUA was 14.71% in the elderly community-dwelling population. The *ABCG2* rs2231142 risk T allele was associated with HUA risk (odds ratio (OR) = 1.63, 95% confidence interval (CI): 1.27–2.11; *p* = 1.65 × 10^− 4^) and with increased sUA levels (Beta = 0.16, *p* = 6.75 × 10^− 9^) in the whole study population. Linear regression analysis showed that the mean sUA level increased linearly with the number of risk alleles of the three candidate genetic variants (Beta = 0.18, *p* = 1.94 × 10^− 12^) The joint effect of the *ABCG2* rs2231142 T allele and cardiovascular risk factors (obesity, hypertension and dyslipidemia) was also associated with increased HUA risk and sUA levels. Each copy of the risk T allele was significantly associated with enhanced HUA risk in patients with hypertriglyceridemia (OR = 2.52, 95% CI: 1.33–4.60; *p* = 0.003) compared to controls.

**Conclusion:**

Our findings reinforce the importance of the *ABCG2* rs2231143 variant as a crucial genetic locus for HUA in Chinese populations and demonstrated the combined effects of multiple genetic risk variants and cardiovascular risk exposures on HUA risk and increased sUA level.

## Background

Hyperuricemia (HUA) is defined by high serum uric acid (sUA) levels due to an imbalance of purines from food, urate synthesis, and/or urate excretion into the urine or gastrointestinal tract [1], which is related to gout, diabetes, hypertension, cardiovascular disease, and kidney disease [1–3]. The cause of HUA is multi-factorial, including age, gender, obesity, diet, alcohol consumption, insulin resistance, hypertension, and medication [1, 4]. sUA, however, is under strong genetic control, and more than ten sUA associated genes have been identified in genome-wide association studies (GWAS) [5, 6], including three main risk genes: ATP-binding cassette subfamily G member 2 (*ABCG2*), glucose transporter type 9 (*GLUT9*, also known as *SLC2A9*), and urate anion transporter 1 (*URAT1*, also known as *SLC22A12*).

*ABCG2* encodes a high-capacity urate efflux transporter [5, 7]. A single nucleotide polymorphism (SNP) rs2231142 (Q141K) located in exon 5 of *ABCG2* leads to a Glu141Lys amino acid substitution [7, 8]. The T allele of rs2231142 has been associated with increased serum urate levels, and its frequency is approximately 3-fold higher in East Asian populations compared to European populations [6, 9]. The *ABCG2* rs2231142 SNP is reported to be the most significant genetic variant associated with HUA and gout in East Asia compared to other sUA-risk genes [6, 9]. However, the association between HUA and the *ABCG2* variants interacting with *SLC2A9* and *SLC22A12* has not been investigated in a Chinese population. In addition, few studies have identified an association of lifestyle-related risk factors (smoking, alcohol consumption, Aspirin use) or disease conditions (hypertension, obesity) with *ABCG2* rs2231142-related HUA risk. Therefore, in this study, we determined if SNPs of three major sUA genes (*ABCG2* rs2231142, *SLC2A9* rs3733591, and *SLC22A12* rs893006), individually and/or in combination were associated with HUA risk and sUA level. We also investigated the association between HUA and cardiovascular risk factors within the *ABCG2* risk allele carriers, to better understand interactions of gene-cardiovascular risk factors and to develop personalized treatment strategies for *ABCG2* rs2231142-related HUA.

## Methods

### Ethics statement

This study was approved by the Ethics Committee of Xuanwuu Hospital (Beijing, China) and all participants signed informed consent before enrolling into this study.

### Study design and subjects

Subjects were recruited from the Beijing Community-dwelling Study on Aging cohort between 2015 and 2016. The study was designed to explore the health status in older adults. Using a stratified multiphase sampling design, a total of 1260 individuals who included representative samples from the urban district in Beijing were enrolled. Subjects with missing sUA data (*n* = 5) or DNA sequencing data (*n* = 30), as well as history of severe chronic kidney disease [endogenous creatinine clearance (Ccr) < 30 ml/min] (*n* = 19) were excluded. To ensure that estrogen did not influence sUA levels in females, we recruited both males (*n* = 699) and females (*n* = 507) who were ≥ 65 years old. HUA was defined as a serum urate acid level > 417 μmol/L (7.0 mg/dl) in males and > 357 μmol/L (6.0 mg/dl) in females according to a previous study [10]. The criteria for the controls included individuals without history and/or current treatment of HUA or gout, and sUA levels of ≤417 (7.0 mg/dl) in males and ≤ 357 μmol/L (6.0 mg/dl) in females.

### Collection of laboratory test and physical examination data

Venous blood was collected from each subject in the morning after fasting for at least 12 h. Samples were analyzed for fasting blood glucose (FBG), triglyceride (TG), low-density lipoprotein (LDL), high-density lipoprotein (HDL) levels, and sUA using IPE (www.ipe-bio.com) in Beijing. sUA levels were assessed using uricase/peroxidase methods. Resting blood pressure (BP) was measured by well-trained personnel using the auscultatory method and the phase I and V (sudden reduction/disappearance) Korotkoff sounds to identify systolic BP (SBP) and diastolic BP (DBP), respectively. Each participant was seated comfortably in a quite environment for 5 min before BP measurements. BP levels were measured in both upper arms at the first office visit to detect possible difference between arms, and the arm with the higher BP value was used for subsequent measurements with an appropriate cuff size for the arm circumference. Three BP measurements were recorded within 1–2 min of each measurement and the average of the three measurements was used for data analysis. The office hypertension was defined as office SBP ≥ 140 mmHg and/or DBP ≥ 90 mmHg in any individual, and subjects who had been receiving antihypertensive medication were also defined as hypertensive [11]. Diabetes mellitus was diagnosed based on the guidelines of the American Diabetes Association (ADA). Body mass index (BMI) was calculated as weight (kg) / height (m^2^) and obesity was defined as BMI ≥ 24 kg/m^2^. High levels of TG and LDL were defined as ≥1.7 mmol/L and ≥ 3.4 mmol/L, respectively, and low HDL levels were defined as < 1.0 mmol/L according to a previous study [12]. Other lifestyle information, such as tobacco smoking and alcohol consumption, was recorded through face-to-face questionnaires.

### SNP selection and genotyping

Three SNPs, including *ABCG2* rs2231142, *SLC2A9* rs3733591, and *SLC22A12* rs893006, were selected based on previously reported associations with sUA levels, especially in Asian populations [8, 13–15]. The minor allele frequencies (MAF) of these selected SNPs were ≥ 5% in Chinese populations according to the 1000 Genomes Projects (http://www.internationalgenome.org/) database.

Genomic DNA was extracted from peripheral blood samples of each participant using a standard procedure, genotyped for the three SNPs using the TaqMan assay (Applied Biosystems, Foster City, CA), and then analyzed for allelic discrimination using the ABI PRISM 7900 Sequence Detection System and SDS software (Applied Biosystems).

### Statistical analysis

Continuous variables are summarized as mean ± standard deviation (SD) and were analyzed using Student’s *t*-test for normal data distributions or the Mann-Whitney *U* test for non-normal data distributions. Comparisons of categorical variables were analyzed using the Chi-square test. The association of risk allele frequencies of these three loci with HUA was analyzed based on gender. The association between the risk allele of three loci and sUA level or HUA was analyzed using the linear regression model and logistical model, respectively, with justification for age, gender, hypertension, serum creatinine, BMI, TG, HDL, and LDL. The association of three SNP genotypes and sUA levels was analyzed using the one-way ANOVA with a posthoc Bonferroni test and a Bonterroni correction of *p* < 0.0167 (equivalent to *p* < 0.05 significance). The genetic risk score of individuals was generated by counting the number of risk alleles of high sUA levels in the study population, ranging from 0 to 6 (C allele of *SLC2A9* rs3733951, T allele of *ABCG2* rs2231142, and G allele of *SLC22A12* rs893006). The association between genetic risk score and sUA levels was analyzed using a linear regression model after adjusting for the above listed covariates. To assess the effects of cardiovascular risk factors on the risk of HUA associated with the *ABCG2* T allele, multiple logistic regression analysis was also conducted. Odds ratios (OR) and 95% confidence intervals (95% CI) were then calculated. All data were analyzed using SPSS version 17.0 (SPSS, Chicago, IL, USA), and a *p* value < 0.05 was considered statistically significant.

## Results

### Characteristics of study population

A total of 1026 individuals, including 699 males and 507 females, were enrolled in this study. The average age of the participants was 76.64 ± 6.40 years (range, 65–94 years) and all were from an elderly Beijing community-dwelling population. The prevalence of HUA was 14.71% in the study population, with 151 HUA cases and 1055 controls. Table 1 indicates that individuals in the HUA group had higher levels of serum creatinine, TG, LDL, and BMI, but lower levels of HDL, compared to controls (*p* < 0.05). The percentage of hypertensive patients in the HUA cases was also higher compared to the control group (47.7% vs 38.8%, *p* = 0.041), but there was no significant difference in SBP and DBP between the two groups, which may be related to hypertension treatment (e.g., 31.8% of the HUA cases and 18.8% of the controls, *p* = 4.9 × 10^− 5^). However, the ratio of diuretics was relatively low and did not reach statistical significance between the two groups. Moreover, there was no significant difference in FBG, alcohol consumption, tobacco smoking, or history of diabetes between the case and control groups.

**Table 1:** Basic clinical and biochemical characteristics of participants

Variables	Hyperuricemia *n* = 151	Control *n* = 1055	*p*-value
sUA (μmmol/L)	441.74 ± 53.53	290.31 ± 60.81	–
Age	77.01 ± 6.90	76.59 ± 6.32	0.66
Male (n, %)	78 (51.7)	621 (58.9)	0.095
TG (mmol/L)	2.07 ± 1.26	1.43 ± 0.84	8.10 × 10^−14^
LDL (mmol/L)	3.47 ± 0.78	3.22 ± 0.81	0.001
HDL(mmol/L)	1.24 ± 0.30	1.35 ± 0.32	2.22 × 10^− 5^
FBG (mmol/L)	6.01 ± 1.50	6.05 ± 1.88	0.31
Serum creatinine (μmol/L)	72.25 ± 12.24	68.83 ± 13.19	0.003
BMI (kg/m^2^)	26.85 ± 2.34	24.14 ± 2.98	1.01 × 10^− 26^
Current drinking (%)	30 (19.9)	248 (23.5)	0.35
Current smoking (%)	34 (22.5)	255 (24.2)	0.68
Office SBP (mmHg)	129.34 ± 15.65	127.71 ± 11.61	0.84
Office DBP (mmHg)	78.54 ± 8.66	79.20 ± 11.69	0.25
Hypertension (%)	72 (47.7)	409 (38.8)	0.041
Hypertension treatment	48 (31.8)	198 (18.8)	4.90 × 10^−5^
Diuretic (%)	6 (4.0)	42 (4.0)	0.99
Diabetes (%)	30 (19.9)	192 (18.2)	0.65

### Distribution of *ABCG2*, *SLC2A9*, and *SLC22A12* polymorphisms between two groups

The three tested SNPs (*SLC2A9* rs3733591, *ABCG2* rs2231142, and *SLC22A12* rs893006) were in Hardy-Weinberg equilibrium (*p* = 0.30, 0.70, and 0.33, respectively). The genotype and allele frequencies of these three SNPs are shown in Table 2 and stratified by gender. In the general population, the genotype frequencies of *ABCG2* rs2231142 among HUA cases were 11.9% (T/T), 53.6% (T/G), and 34.4% (G/G), respectively, while the genotype frequencies were 8.0% (T/T), 40.4% (T/G), and 51.7% (G/G) in the controls, respectively. The minor T allele of the *ABCG2* variants reached 28.2% in the controls and 38.7% in the HUA cases. The distribution of *ABCG2* variants showed statistical significance in males and when combining males and females for the Chi-square test, there was no significant association in females (*p* = 0.053). The distribution of *SLC2A9* rs3733591 was only significantly different in females (*p* = 0.003), with a frequency of 17.8% for C/C and 52.1% for C/T in the HUA cases. There was no significant difference in genotype frequency of *SLC22A12* rs893006 between HUA cases and controls in both males and females.

**Table 2:** Distribution of three SNPs in HUA cases and controls, stratified by gender

Gene/SNP	Male		Female		Total	
	Case (%) (*n* = 78)	Control (%) (*n* = 621)	Case (%) (*n* = 73)	Control (%) (*n* = 434)	Case (%) (*n* = 151)	Control (%) (*n* = 1055)
SLC2A9 rs3733591						
C/C	10 (12.8)	72 (11.6)	13 (17.8)	33 (7.6)	23 (15.2)	105 (10.0)
C/T	34 (43.6)	284 (45.7)	38 (52.1)	198 (45.6)	72 (47.4)	482 (45.7)
T/T	34 (43.6)	265 (42.7)	22 (30.1)	203 (46.8)	56 (37.1)	468 (44.4)
*p*^a^	0.91		0.003		0.075	
Allele (%)						
C	54 (34.6)	428 (34.5)	604 (69.6)	264 (30.4)	118 (39.1)	692 (32.8)
T	102 (65.4)	814 (65.5)	82 (56.2)	64 (43.8)	184 (60.9)	1418 (67.2)
ABCG2 rs2231142						
T/T	8 (10.3)	50 (8.1)	10 (13.7)	34 (7.8)	18 (11.9)	84 (8.0)
T/G	46 (59.0)	253 (40.7)	35 (47.9)	173 (39.9)	81 (53.6)	426 (40.4)
G/G	24 (30.8)	318 (51.2)	28 (38.4)	227 (52.3)	52 (34.4)	545 (51.7)
*p*^a^	0.003		0.053		6.37 × 10^−5^	
Allele (%)						
T	62 (39.7)	353 (28.4)	55 (37.7)	241 (27.8)	117 (38.7)	594 (28.2)
G	94 (60.3)	889 (71.6)	91 (62.3)	627 (72.2)	185 (61.3)	1516 (71.8)
SLC22A12 rs893006						
G/G	51 (65.4)	346 (55.7)	39 (53.4)	218 (50.2)	90 (59.6)	564 (53.5)
G/T	25 (32.1)	234 (37.7)	31 (42.5)	187 (43.1)	56 (37.1)	421 (39.9)
T/T	2 (2.6)	419 (6.6)	3 (4.1)	29 (6.7)	5 (3.3)	70 (6.6)
*p*^a^	0.16		0.67		0.17	
Allele (%)						
G	127 (81.4)	926 (74.6)	109 (74.7)	623 (71.8)	561 (26.6)	1549 (73.4)
T	29 (18.6)	316 (25.4)	37 (25.3)	245 (28.2)	66 (21.9)	236 (78.1)

^a^Analyzed by using the χ^2^ test for association between genotypes of each SNP and HUA

### Association between risk alleles of uric acid genes with sUA levels and HUA risk

In all samples, there was a significant association of increased sUA levels (μmol/L) with each copy of risk allele of the three SNPs (Beta = 0.08, *p* = 0.002 in rs3733591 *SLC2A9*, Beta = 0.16, *p* = 6.75 × 10^− 9^ in rs2231142 *ABCG2*, Beta = 0.07, *p* = 0.006 in rs893006 *SLC22A12*; Table 3). There was also a significant association HUA with the risk alleles of these three SNPs [OR (95% CI): 1.34 (1.03–1.74), *p* = 0.029 in rs3733591 *SLC2A9,* OR (95% CI): 1.63 (1.27–2.11), *p* = 1.65 × 10^− 4^ in rs2231142 *ABCG2*]; however, there was no significant association of HUA with *SLC22A12* rs893006 variants [OR (95% CI): 1.33 (0.99–1.78), *p* = 0.061]. After we separated the subjects by gender, the T allele of rs2231142 *ABCG2* was associated with sUA level and HUA in both males and females (all *p* < 0.05), but the C risk allele in rs3722591 *SLC2A9* was only associated with sUA level and HUA in females. The *SLC22A12* rs893006 G risk allele was only associated with sUA level in males.

**Table 3:** Association of risk alleles of uric acid genes with sUA levels and HUA

Gene	SNP	Coded/other allele	CAF (%)	sUA level		Hyperuricemia	
				Beta (SE)	*p*^a^	OR (95%CI)	*p*^b^
Total							
*SLC2A9*	rs3733591	C/T	33.58	0.08 (3.02)	0.002	1.34 (1.03–1.74)	0.029
*ABCG2*	rs2231142	T/G	29.48	0.16 (3.98)	6.75 × 10^−9^	1.63 (1.27–2.11)	1.65 × 10^− 4^
*SLC22A12*	rs893006	G/T	74.00	0.07 (3.25)	0.006	1.33 (0.99–1.78)	0.061
Male							
*SLC2A9*	rs3733591	C/T	32.35	0.05 (3.96)	0.122	1.09 (0.72–1.64)	0.699
*ABCG2*	rs2231142	T/G	29.69	0.16 (4.15)	4.48 × 10^−7^	1.70 (1.20–2.42)	0.003
*SLC22A12*	rs893006	G/T	75.32	0.08 (4.34)	0.018	1.49 (0.98–2.28)	0.064
Female							
*SLC2A9*	rs3733591	C/T	34.48	0.13 (4.68)	0.002	1.89 (1.26–2.85)	0.002
*ABCG2*	rs2231142	T/G	29.19	0.17 (4.68)	6.56 × 10^−5^	1.60 (1.10–2.32)	0.013
*SLC22A12*	rs893006	G/T	72.19	0.06 (4.90)	0.140	1.17 (0.75–1.84)	0.489

*CAF* code allele frequency

^a^Liner regression adjusted by age, gender, BMI, hypertension, serum creatinine, TG, HDL, and LDL

^b^Logistic regression adjusted by age, gender, BMI, hypertension, serum creatinine, TG, HDL, and LDL

### Association between different genotypes of three SNPs and sUA level

sUA level (μmol/L) was associated with the *ABCG2* rs2231142 polymorphism in all populations (T/T: 337.46 ± 71.25, T/G: 317.51 ± 80.53, G/G: 287.46 ± 75.13; *p* = 6.98 × 10^− 8^) and sUA levels of the G/G genotypes were significantly lower compared with those of the T/T genotypes (*p* = 4.40 × 10^− 6^). There was also a significant difference in sUA levels in the G/G and T/T male carriers (G/G vs. T/T: 313.32 ± 73.83 vs 348.20 ± 68.85; *p* = 0.003) and female carriers (G/G vs T/T: 276.18 ± 71.65 vs 323.32 ± 72.67; *p* = 2.67 × 10^− 4^). The sUA level was higher in *SLC2A9* C/C carriers of the whole population and females compared with T/T carriers (*p* = 0.005 and 0.015, respectively), but there was no association in male subjects. There was also no significant difference in sUA levels between each genotype of the *SLC22A12* rs893006 polymorphism analyzed using the posthoc Bonferroni test (Table 4).

**Table 4:** Association between genotypes of three SNPs and serum urate levels in the study population, separated by gender

Gene (SNP)	Code /other allele (1/2)	SUA (mean ± SD) μmol/L			*p*^a^	*p*^b^	*p*^b^	*p*^b^
		1/1	1/2	2/2		1/1 vs 1/2	1/2 vs 2/2	1/1 vs 2/2
SLC2A9 rs3733951	C/T							
Total		327.24 ± 82.39	311.09 ± 77.71	302.96 ± 76.89	0.005	0.262	0.104	0.005
Male		337.71 ± 72.96	326.42 ± 78.23	324.04 ± 78.28	0.356	–	–	–
Female		308.57 ± 94.96	290.43 ± 72.17	274.96 ± 69.89	0.006	0.073	0.379	0.015
ABCG2 rs2231142	T/G							
Total		337.46 ± 71.25	317.51 ± 80.53	287.46 ± 75.13	6.98 × 10^−8^	0.052	5.42 × 10^−5^	4.40 × 10^−6^
Male		348.20 ± 68.85	337.90 ± 78.10	313.32 ± 73.83	1.85 × 10^−5^	1.26 × 10^−4^	1.000	0.003
Female		323.32 ± 72.67	288.21 ± 74.89	276.18 ± 71.65	3.45 × 10^−4^	0.012	0.236	2.67 × 10^−4^
SLC22A12 rs893006	G/T							
Total		315.33 ± 80.50	302.80 ± 75.98	297.60 ± 66.03	0.012	0.023	1.000	0.186
Male		392.11 ± 79.52	320.66 ± 72.85	313.54 ± 63.71	0.087	–	–	–
Female		289.41 ± 75.08	281.58 ± 74.28	276.17 ± 64.37	0.402	–	–	–

^a^ANOVA comparison between groups;

^b^Bonferroni test; 1 = code allele; 2 = other allele; *SNP* single-nucleotide polymorphism, *SUA* serum uric acid

### Association of sUA levels and genetic risk scores

**Fig. 1:**
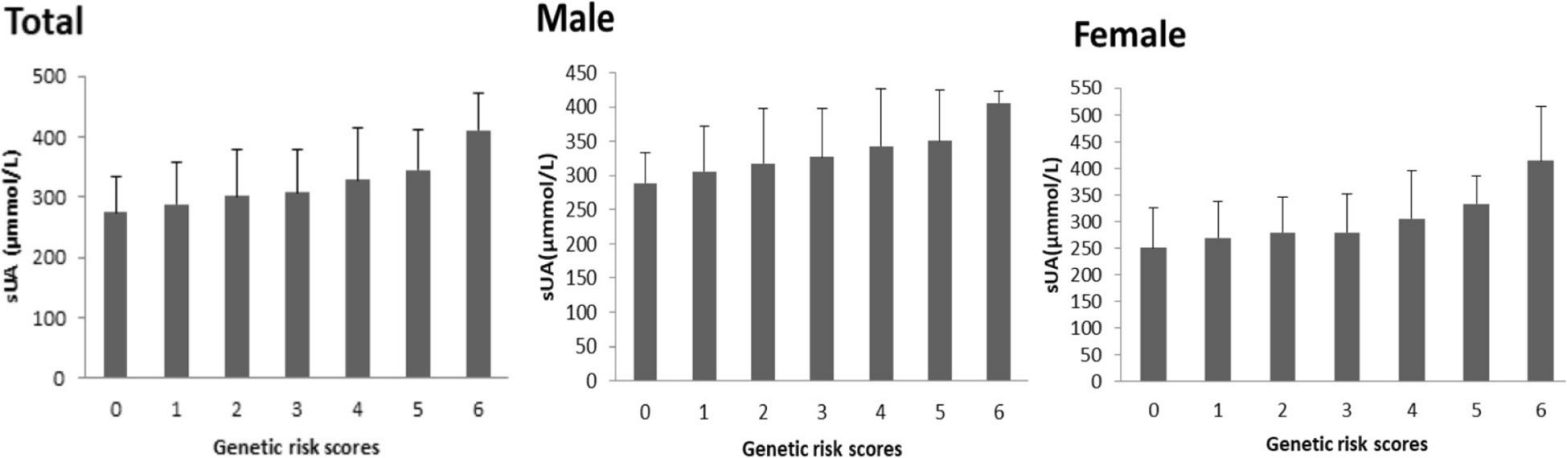
Association of sUA and genetic risk score, constructed with the number of HUA risk alleles, separated by gender (mean sUA and SD for each genetic risk score)

The genetic risk score of an individual was generated by counting the number of risk alleles of high sUA levels in the study population, ranging from 0 to 6 (C allele of *SLC2A9* rs3733951, T allele of *ABCG2* rs2231142, and G allele of *SLC22A12* rs893006; Fig. 1). Using linear regression analysis, we found that the mean sUA level in both males and females significantly increased with the number of risk alleles (Beta = 0.17, *p* = 6.24 × 10^− 7^ in males and Beta = 0.20, *p* = 7.39 × 10^− 7^, respectively). In the whole population, the mean sUA level also increased linearly with the number of risk alleles (Beta = 0.18, *p* = 1.94 × 10^− 12^).

### Association between rs2231142 *ABCG2* variants and HUA risk according to cardiovascular risk factors

Table 5 summarizes the association between *ABCG2* rs2231142 polymorphisms and HUA risk with or without cardiovascular risk factors. In all study populations, the OR of HUA with each copy of the T allele in rs2231142 increased significantly when accounting for cardiovascular risks (such as obesity, hypertension, higher levels of TG and LDL, and lower levels of HDL) compared to those without cardiovascular risk factors. The OR of HUA with high TG levels and low HDL levels had a strong increase in HUA risk associated with each copy of T alleles in 2,231,142 in males (OR (95% CI): 2.41 (1.06–4.53), *p* = 0.035 and OR (95% CI): 2.50 (1.12–5.67), *p* = 0.026, respectively). Among females, the association was statistically significant in individuals with obesity [OR (95% CI): 1.59 (1.04–3.04); *p* = 0.045], hypertension [OR (95% CI): 1.83 (1.09–3.08); *p* = 0.021], and high TG levels [OR (95% CI): 2.57 (1.10–6.01); *p* = 0.030] and LDL levels [OR (95% CI): 1.98 (1.20–3.34); *p* = 0.008] compared with those with non-cardiovascular risk factors, but not those with low HDL levels (*p* = 0.156).

**Table 5:** Association between *ABCG2* rs2231142 variants and HUA according to cardiovascular risk exposure

Cardiovascular risk	Total		Male		Female	
	OR^a^ 95%CI	*p*^b^	OR^a^ 95%CI	*p*^b^	OR^a^ 95%CI	*p*^b^
Obesity status						
BMI < 24 kg/m^2^	1.56 (1.07–2.31)	0.022	1.43 (0.84–2.45)	0.191	1.51 (0.92–2.49)	0.161
BMI ≥ 24 kg/m^2^	1.66 (1.18–2.36)	0.004	1.86 (1.15–3.03)	0.012	1.59 (1.04–3.04)	0.045
Hypertension						
No	1.43 (1.05–2.04)	0.047	1.68 (1.02–2.78)	0.043	1.40 (0.81–2.37)	0.219
Yes	1.88 (1.30–2.71)	0.001	1.91 (1.13–3.24)	0.015	1.83 (1.09–3.08)	0.021
Hyper TG						
TG < 1.7 mmol/L	1.53 (1.14–2.07)	0.005	1.58 (1.04–2.83)	0.031	1.51 (0.98–2.33)	0.060
TG ≥ 1.7 mmol/L	2.52 (1.33–4.60)	0.003	2.41 (1.06–4.53)	0.035	2.57 (1.10–6.01)	0.030
Hyper LDL						
LDL < 3.4 mmol/L	1.43 (1.01–2.02)	0.043	1.68 (1.08–2.62)	0.021	1.18 (0.66–2.09)	0.582
LDL ≥ 3.4 mmol/L	1.90 (1.39–2.79)	0.001	1.75 (1.12–4.80)	0.024	1.98 (1.20–3.34)	0.008
Low HDL						
HDL ≥1.0 mmol/L	1.57 (1.18–2.09)	0.002	1.58 (1.06–2.34)	0.024	1.61 (0.93–2.43)	0.087
HDL < 1.0 mmol/L	2.21 (1.21–4.01)	0.010	2.50 (1.12–5.67)	0.026	1.92 (0.78–4.74)	0.156

^a^ increased odds of HUA associated with each copy of the T allele in ABCG2 rs2231142

^b^*p* value for the logistic regression adjusted by age, gender, serum cretinine, BMI, hypertension, TG, LDL, and HDL

We further assessed the association between cardiovascular risk factors and HUA risk or sUA levels among the *ABCG2* rs2231142 T allele carriers (Table 6). BMI and TG levels were associated with higher sUA levels (Beta = 0.21, *p* = 3.40 × 10^− 8^ and Beta = 0.24, *p* = 6.18 × 10^− 9^, respectively) in the whole population, and this association was also significant in males (Beta = 0.23, *p* = 1.14 × 10^− 5^ for BMI and Beta = 0.25, *p* = 8.95 × 1 0^− 6^ for TG level, respectively) and females (Beta = 0.20, *p* = 0.002 for BMI and Beta = 0.24, *p* = 4.00 × 10^− 4^ for TG level, respectively). The association of BMI and TG level with HUA risk was also significant in males [OR (95% CI) = 2.01 (1.62–2.50); *p* = 1.75 × 10^− 10^ and OR (95% CI) = 1.88 (1.28–2.74); *p* = 0.001*,* respectively] and females [OR (95% CI): 1.22 (1.08–1.37); *p* = 0.001and OR (95% CI): 1.95 (1.26–2.55); *p* = 0.003*,* respectively]. However, other cardiovascular risk factors were not associated with sUA level and HUA risk among rs2231142 T allele carriers.

**Table 6:** Association between cardiovascular risk and sUA concentration and HUA risk among *ABCG2* T allele carriers

Cardiovascular risk factors	sUA		hyperuricemia	
	Beta (SE)	p*	OR 95%CI	p*
Total population				
BMI	0.21 (0.60)	3.40 × 10^−8^	1.42 (1.28–1.56)	4.25 × 10^−12^
Hypertension	0.036 (6.01)	0.978	1.35 (0.85–2.13)	0.206
TG	0.24 (3.67)	6.18 × 10^−9^	1.90 (1.44–2.50)	4.08 × 10^−6^
LDL	0.025 (3.77)	0.506	1.28 (0.94–1.74)	0.114
HDL	−0.042 (10.76)	0.311	0.69 (0.25–1.90)	0.471
Male				
BMI	0.23 (1.27)	1.14 × 10^−5^	2.01 (1.62–2.50)	1.75 × 10^−10^
Hypertension	0.022 (7.83)	0.657	1.37 (0.74–2.54)	0.319
TG	0.25 (4.72)	8.95 × 10^−6^	1.88 (1.28–2.74)	0.001
LDL	0.015 (5.27)	0.761	1.40 (0.84–2.32)	0.192
HDL	−0.063 (14.92)	0.264	0.52 (0.11–2.41)	0.407
Female				
BMI	0.20 (1.48)	0.002	1.22 (1.08–1.37)	0.001
Hypertension	0.054 (9.45)	0.370	1.27 (0.63–2.55)	0.498
TG	0.24 (5.93)	4.00 × 10^−4^	1.95 (1.26–3.03)	0.003
LDL	0.073 (5.44)	0.231	1.30 (0.87–1.41)	0.208
HDL	0.025 (15.72)	0.711	0.83 (0.19–3.70)	0.798

*p value for the logistic regression adjusted by age, gender, serum cretinine, BMI, hypertension, TG, LDL, and HDL

## Discussion

In the current study, we confirmed that *ABCG2* rs2231142 is the most important sUA genetic variant compared to two other sUA genetic polymorphisms, *SLC2A9* rs3733591 and *SLC22A12* rs893006, in the Chinese population. Our data also showed that the combination of the *ABCG2* rs2231142 risk allele with two other sUA genetic variants was significantly associated with an increase in sUA level in an elderly Chinese population. Our data are consistent with findings reported by Torres et al. [16], although the studies included different SNPs for *SLC2A9* and *SLC22A12*. In a single-locus analysis, the *SLC22A12* SNP rs893006 was reported to be associated with HUA in Japanese men [13]. This locus was related to sUA levels only in males, but there was no significant association with HUA in males or females in the present study, which was in accordance with the findings of a previous study [15]. SNP rs3733591 in *SLC2A9* was also identified as being associated with sUA levels and gout in the Han Chinese population [14], but the association was only significant in females in our study. However, the combined effect of the alleles on sUA level was enhanced for carriers of these genetic variants, which indicates that three urate transporter genes interact in the cortical cells in renal tubules. Although our study should be validated with a larger cohort, our findings revealed that assessing the combined effect of genetic factors is more valuable for developing a treatment strategy based on inter-individual differences, rather than merely testing for single genotype variants to predict HUA or gout risk.

Genetic factors play an important role in HUA risk, but these factors do not change in a given individual. Thus, our results further demonstrated that cardiovascular risk factors, such as obesity, hypertension, and dyslipidemia, combined with the *ABCG2* rs2231142 risk allele, greatly modified the risk of HUA and increased sUA levels. Most importantly, we revealed that *ABCG2* rs2231142 T allele carriers with high TG levels have a significantly increased risk of HUA and sUA levels. Associations with sUA levels and lipid metabolism were not clearly reported in a previous study [17], but Fu et al reported that TG, LDL, and HDL levels were significantly associated with HUA risk (OR = 1.17, 1.20, and 0.62, respectively) in a Chinese community-dwelling population [2]. Another Chinese prospective study suggested that TG level was a significant and independent risk factor for HUA [4]. The associations found in our study were greatly higher than those reported in previous Chinese studies, possibly due to the older population included in our study. Our findings suggest that more attention should be given to controlling serum TG levels and that appropriate diet recommendations or lipid-lowering therapy should be provided to elderly Chinese patients, given the high frequency of the *ABCG2* rs2231142 risk allele among Asians.

The association of hypertension with HUA risk has been inconsistently reported in the literature [2, 8]. In our current study, HUA risk was associated with hypertension, but not with DBP or SBP measured at office visits, which could be mainly due to BP measurements that were possibly affected by anti-hypertension medicine. Furthermore, the proportion of diuretic intake in this the cohort of participations was low and there was no difference between HUA cases and controls; thus, the influence on sUA should be relatively small. Our study also established that HUA risk was increased in *ABCG2* rs2231142 risk allele carriers in the hypertensive patients compared to non-hypertension patients. A previously published rodent model demonstrated that hypertension could induce renal arteriosclerosis and tubulointerstitial disease, leading to reduced uric acid excretion and HUA [18]. Therefore, the molecular mechanisms underlying the association remain to be determined.

A previous study using a mouse model showed that *ABCG2* expression increased significantly, suggesting a link between enhanced urate reabsorption and obesity-associated HUA [19]. Our current data further confirmed that both obese males and females carrying the rs2231142 T allele has an associated increased HUA risk, which was consistent with other Asian population studies [8, 20]. However, a previous study in a European population only showed a significant association in males, not in females [21], possibly due to ethnic differences. Estrogen levels can affect *ABCG2* expression [22, 23], thus we excluded postmenopausal women to eliminate the effect of estrogen on allelic associations with HUA.

Our current study does have some limitations. For example, we did not include all cardiovascular risk factors that could impact sUA levels and HUA risk, such as lifestyle and dietary factors. In addition, more samples from different districts need to be included in future analyses to better investigate the association of *ABCG2* polymorphisms and other genetic variants and cardiovascular risk factors with the risk of developing HUA.

## Conclusions

Our current study demonstrated that the *ABCG2* rs2231142 variant in combination with the *SLC2A9* and *SLC22A12* genetic variants significantly increased sUA levels in a Chinese community population. The *ABCG2* rs2231142 variant in combination with cardiovascular risk factors, specifically higher TG levels, is significantly associated with increased sUA levels and HUA risk.

## Data Availability

According to the Chinese policy of “National regulation on the management of human genetic resources” released by State Council (Index No. 000014349/2019–00063; Serial No. 171), availability of the data and materials is confidential.

## Abbreviations

ABCG2: ATP-binding cassette subfamily G member 2
BMI: Body mass index
Ccr: Creatinine clearance
DBP: Diastolic blood pressure
FBG: Fasting blood-glucose
GLUT9: Glucose transporter type 9
GWAS: Genome-wide association studies
HDL: High density lipoprotein
LDL: Low-density lipoprotein
MAF: Minor allele frequencies
SBP: Systolic blood pressure
SD: Standard deviation
SNP: Single nucleotide polymorphism
sUA: serum uric acid
TG: Triglyceridemia
URAT1: Urate anion transporter 1
